# PPGV: a comprehensive database of peach population genome variation

**DOI:** 10.1186/s12870-024-05437-2

**Published:** 2024-07-24

**Authors:** Yanlin An, Qiuyan Ban, Li Liu, Feng Zhang, Shirui Yu, Tingting Jing, Shiqi Zhao

**Affiliations:** 1 Department of Food Science and Engineering, Moutai Institute, Renhuai, China; 2 School of Fishery, Zhejiang Ocean University, Zhoushan, Zhejiang 316022 China; 3 State Key Laboratory of Tea Plant Biology and Utilization, Anhui Agricultural University, Hefei, China; 4 College of Horticulture, Henan Agricultural University, Zhengzhou, China

**Keywords:** Peach tree, Genome, Variation, Database, Tools module

## Abstract

Peach tree is one of the most important fruit trees in the world, and it has been cultivated for more than 7,500 years. In recent years, the genome and population resequencing of peach trees have been published continuously, which has effectively promoted the research of peach tree genetics and breeding. In order to promote the further mining and utilization of these data, we integrated and constructed a comprehensive peach genome and variation database (PPGV, http://peachtree.work/home). The PPGV contains 10 sets of published peach tree genome data, as well as genomic variation information for 1,378 peach tree samples (the resequencing data of 1,378 samples were aligned with the high-quality genomes of Lovell, CN14 and Chinesecling, respectively, for mutation detection). A variety of useful and flexible tools, such as BLAST, Gene ID Convert, KEGG/GO Enrichment, Primer Design and Gene function, were also specially designed for searching data and assisting in breeding.

## Introduction

Peach tree (*Prunus persica*) is one of the most representative and widely cultivated crops of the rose family that originated in China [1]. Its fruit has a wonderful flavor and rich nutrients, thus giving the peach fruit high economic and health value. Peach trees were already planted in China at least 7,500 years ago and spread to most of the world over the millennia since. Today, peach is the fourth largest fruit after apples, pears and grapes, with 1,527,052 hectares harvested worldwide [2, 3].

The advancement of sequencing technology and the rapid reduction of sequencing costs have greatly promoted the research process of plant genome and population evolution. Up to now, more than 1,300 plant genomes have been published and are still growing rapidly (https://www.plabipd.de/index.ep). For peach trees, at least five more peach tree genomes have been released since the first genome was published in 2013 [1, 2, 4–6]. Studies have shown that the genomes of different peach tree varieties have significant differences, such as “LHSM” and “124 Pan” with genome sizes of 257.2 Mb and 206 Mb, respectively [4, 6]. To reveal the genetic diversity and evolutionary history of peach trees, many population resequencing studies have been widely carried out based on the above reference genomes. For example, Cao et al. [7] identified 409 selected genes based on resequencing analysis of 10 wild and 74 cultivated peach, and proved that human breeding activities led to a sharp decline in the heterozygosity proportion of SNPs; Guan et al. [8] identified a 1.67 Mb structural variation controlling the shape of peach fruit through genome and resequencing analysis of 149 peach trees; Li et al. [9] and Yu et al. [6] analyzed the domestication patterns and the biochemical basis of fruit flavor in the breeding process of peach trees through resequencing data analysis of 480 and 564 peach accessions, respectively. Extensive large-scale resequencing not only greatly promoted the research progress of peach trees, but also released a large amount of raw sequencing data. Effectively integrating and utilizing these data is undoubtedly quite challenging [10, 11].

For many food and cash crops, various functional databases have been successfully constructed [12–14]. For example, Gui et al. [15] constructed a comprehensive maize multiomics database; based on thousands of soybean germplasm resources, Zheng et al. [16] built a large database containing 65,374,688 SNPs and 10,952,749 InDels. In addition, databases of genetic variation of species such as *Brassica napus*, sorghum and tea plants have been successfully published [17–19]. In the previous research, although some databases of *Rosaceae* plants have been reported (https://www.rosaceae.org/), the database dedicated to the genetic variation of peach tree population has not been constructed.

## Materials and methods

### Retrieval of genome and resequencing data resources

Before constructing the database, we first obtain peach tree genome sequences, gff files, protein sequences, and a large amount of resequencing data from public databases such as NCBI (https://www.ncbi.nlm.nih.gov/), CNCB (https://www.cncb.ac.cn/) and GDR (https://www.rosaceae.org/). In addition to the ten sets of genomic data (including ‘Lovell’, ‘CN14’, ‘Chinesecling’, ‘Pfe’, ‘Pka’, ‘Pmi’, ‘Pda’, ‘LHSM’, ‘RYP1’, ‘124Pan’), another 1,378 samples of resequencing data were downloaded for analysis. For the resequencing data, detailed information can be found in the Overview section of this database (http://peachtree.work/overview).

### Identification of the peach tree genome and population genetic variation

In this study, we chose the ‘Lovell’ (about 220 Mb), ‘CN14’ (about 222 Mb), and ‘Chinesecling’ (about 236 Mb) peach tree genomes as reference genomes and used the BWA software to build indexes for each of them [19]. The downloaded sequences were filtered using the following parameters of the fastp software: --length_required = 25 --cut_front --cut_window_size 4 --cut_mean_quality 15 [20]. High quality reads of all samples were aligned to the reference genome with BWA software under the default reference. Following that, use the samtools software to convert the SAM file to the BAM format, then sort and build an index. PCR duplicates were removed using the Picard software [21]. Subsequently, the HaplotypeCaller mode of GATK software is used to identify SNP/INDEL mutation sites with default parameters, and finally the original mutation data set is obtained. Due to differences in the depth of resequencing among populations, we used the --minQ parameter to filter SNPs (--minQ 180) and INDELs (--minQ 50) rseparately, and annotated all the mutation sites by snpEFF software [22]. At the same time, structural variations were identified using the default parameters of the LUMPY software [23].

### Genomic and protein sequence analysis

We first used EDTA software to perform a preliminary identification of transposons in each genome, and TEsorter software was used to classify unknown types of transposons based on the above identification results, and then further identification was performed using EDTA software to ensure the reliability of the results [24, 25]. The MISA software was used to identify SSR (Simple Sequence Repeat) loci on different genomes, with the specific definition (unit_size, min_repeats) parameters as follows: 2–6, 3–6, 4–5, 5 − 4, 6 − 4. At the same time, we annotate the protein sequence with eggNOG online website (http://eggnog5.embl.de/#/app/home) and pfamscan software respectively. In addition, transcription factors were further identified and annotated by iTAK website (http://itak.feilab.net/cgi-bin/itak/index.cgi).

### Integration of other biological analysis tools

On this website, we offer several practical bioinformatics tools such as get sequence, blast, primer design, and enrichment analysis. These tools are primarily implemented by utilizing bioinformatics software and R packages such as seqtk, blast, primer3, and clusterProfiler. In addition, some softwares such as ORFfinder, SSRMMD and TBtools are also used for data analysis or to provide support for this site [26, 27].

### Database construction

We used Flask (a web package encoded by Python) and Dash (a fast Python web framework package) to build the basic framework of this website, and used Hypertext Markup Language (HTML) and Cascading Style Sheets (CSS) to refine the web layout. All interactive functions were implemented through the third-party plugin feffery-antd-components, which provides rich UI components and interactive functions to help developers quickly build interactive web applications. At the same time, we also used the cacheout caching technology to enhance the response speed of the website. All the data were imported into the MySQL database to improve the query speed. Finally, the website was deployed on Ubuntu 22.04 LTS system provided by Tencent Cloud.

## Results

### Summary of the PPGV database

**Fig. 1:**
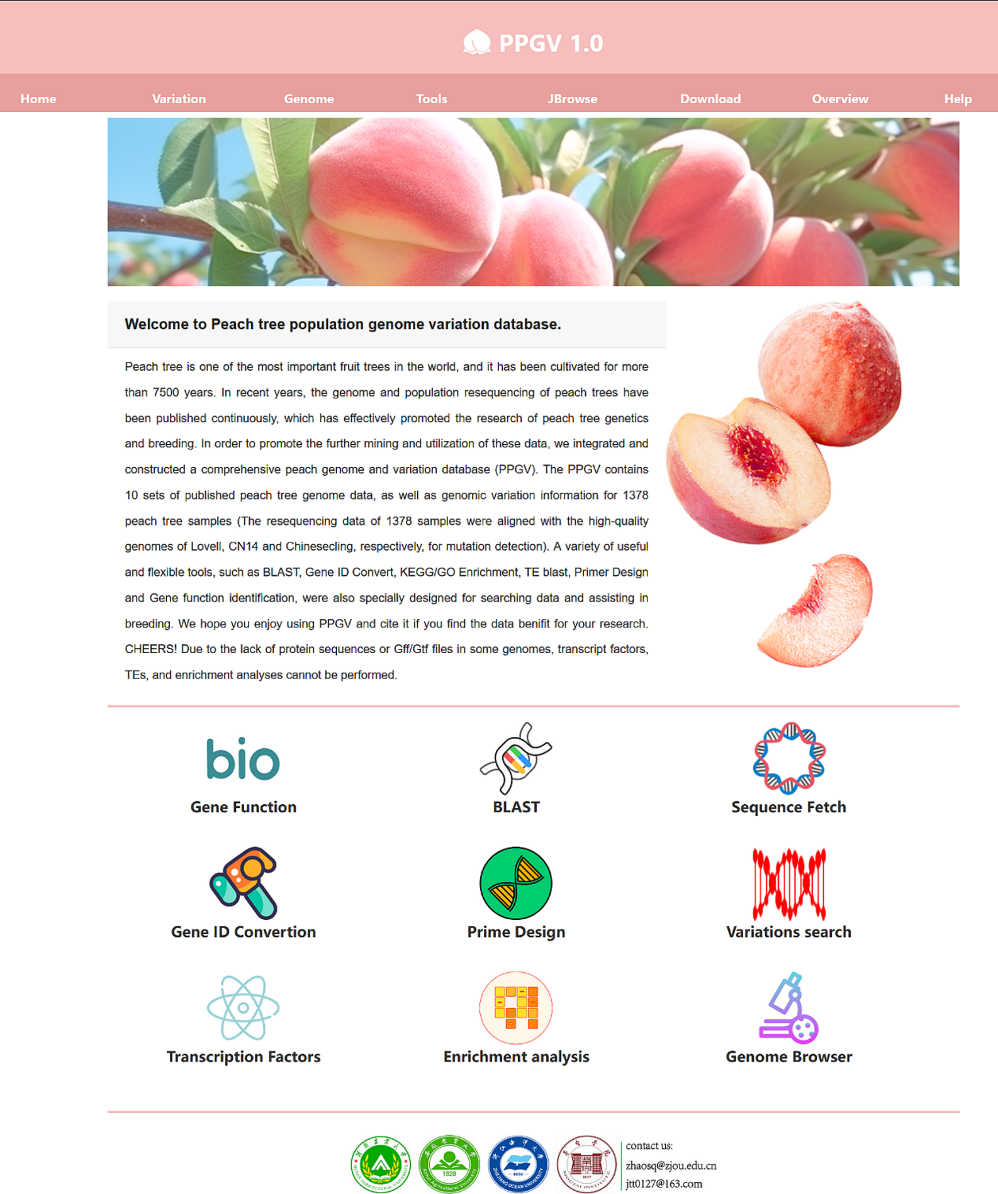
Screenshot of the homepage of PPGV database, showing a subset of available genome sequences, search and analysis tools

In this study, we constructed a comprehensive peach genome and population variation database, which includes genetic variation information for 1,378 peach samples aligned to the “Lovell”, “CN14”, and “Chinesecling” genomes, as well as genome information from 10 publicly available peach genomes. Apart from the Home and Overview pages, PPGV mainly consists of five functional modules: Variation, Genome, JBrowse, Tools, and Download. In the following, we will provide a detailed introduction to these modules (Fig. 1). However, it is particularly important to note that due to some studies only publishing genome sequences without accompanying protein sequences, gff files, or accompanying files that cannot be used, it is difficult to perform transcript factor, collinearity, and other analyses.

### Variation module

**Fig. 2:**
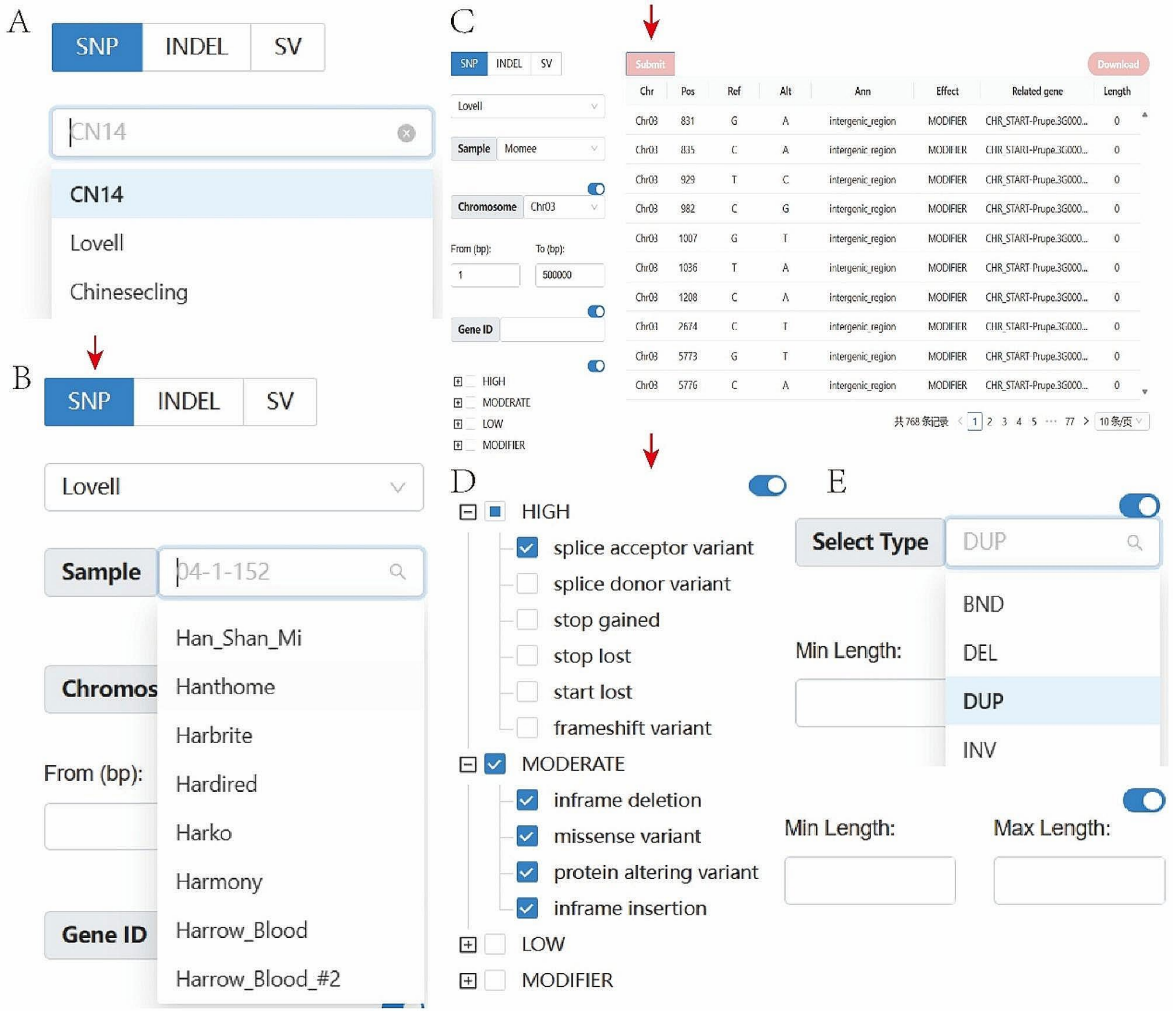
Flow chart of mutation search module. (**A**) Select reference genome. (**B**) Selection of samples. (**C**) Display of search results. (**D**) Search according to the annotation results. (**E**) Filter the results according to mutation type or length

In the current module, users can choose any one of the three reference genomes, “CN14”, “Lovell”, and “Chinesecling”, and then search for SNP, INDEL, or SV variations in different samples (Fig. 2A). According to the different types of mutations, we provide diverse search modes. For SNP and INDEL, after selecting the reference genome, further selection of the sample name and chromosome information is required, and the range interval of the starting and ending positions of the variant site needs to be entered (Fig. 2B). After that, clicking the “Submit” button will submit the query conditions and view the corresponding results (Fig. 2C). At the same time, users can enter the gene ID to further filter the above search results to view specific gene-related mutation sites. They can also query specific types of mutations based on SnpEff annotation results, such as “splice acceptor variant” and “MODERATE” (Fig. 2D). For INDEL and SV, users can also filter the query results based on the mutation type and length (Fig. 2E).

### Genome module

**Fig. 3:**
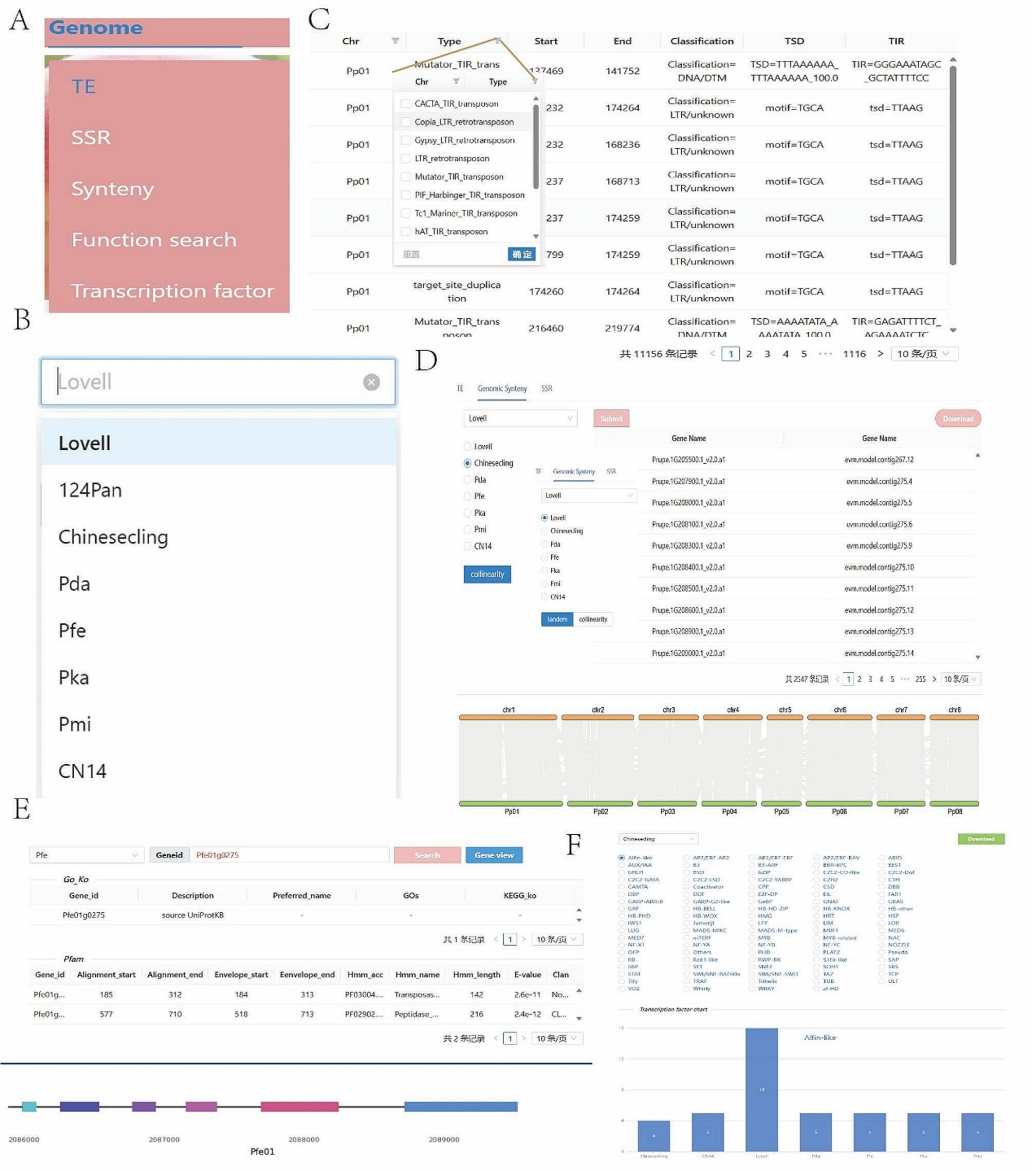
Functional schematic of Genome module. (**A**) Select the submodule. (**B**) Select the genome. (**C**) Example of transposon search results. (**D**) Example plot of tandem repeat and genomic collinearity results (**E**) Examples of gene function search results. (**F**) Transcription factor submodule

In the Genome module, PPGV provides three search functions including TE/SSR/Synteny, Function search, and Transcription factor (Fig. 3A). Taking the TE/SSR/Synteny sub-module as an example, when users enter the interactive interface, they need to select the genome first, and then click the submit button to get the corresponding results (Fig. 3B). Based on the result list, users can further filter the results based on chromosome or the type of transposon/SSR (Fig. 3C). Meanwhile, through the Genomic Synteny function, users can query the tandem duplicated genes within different peach tree genomes and the collinear gene pairs between genomes (Fig. 3D). In the Function search sub-module, users can easily obtain the GO, KEGG and Pfam annotation information of different peach tree genomes, and visualize the gene structure of specific genes (Fig. 3E). In addition, we provide a transcription factor search function. Users can not only quickly download all the sequences of specific transcriptome factors in each genome, but also observe the quantitative characteristics of transcription factors in different peach genomes through column charts (Fig. 3F).

### Tools module

**Fig. 4:**
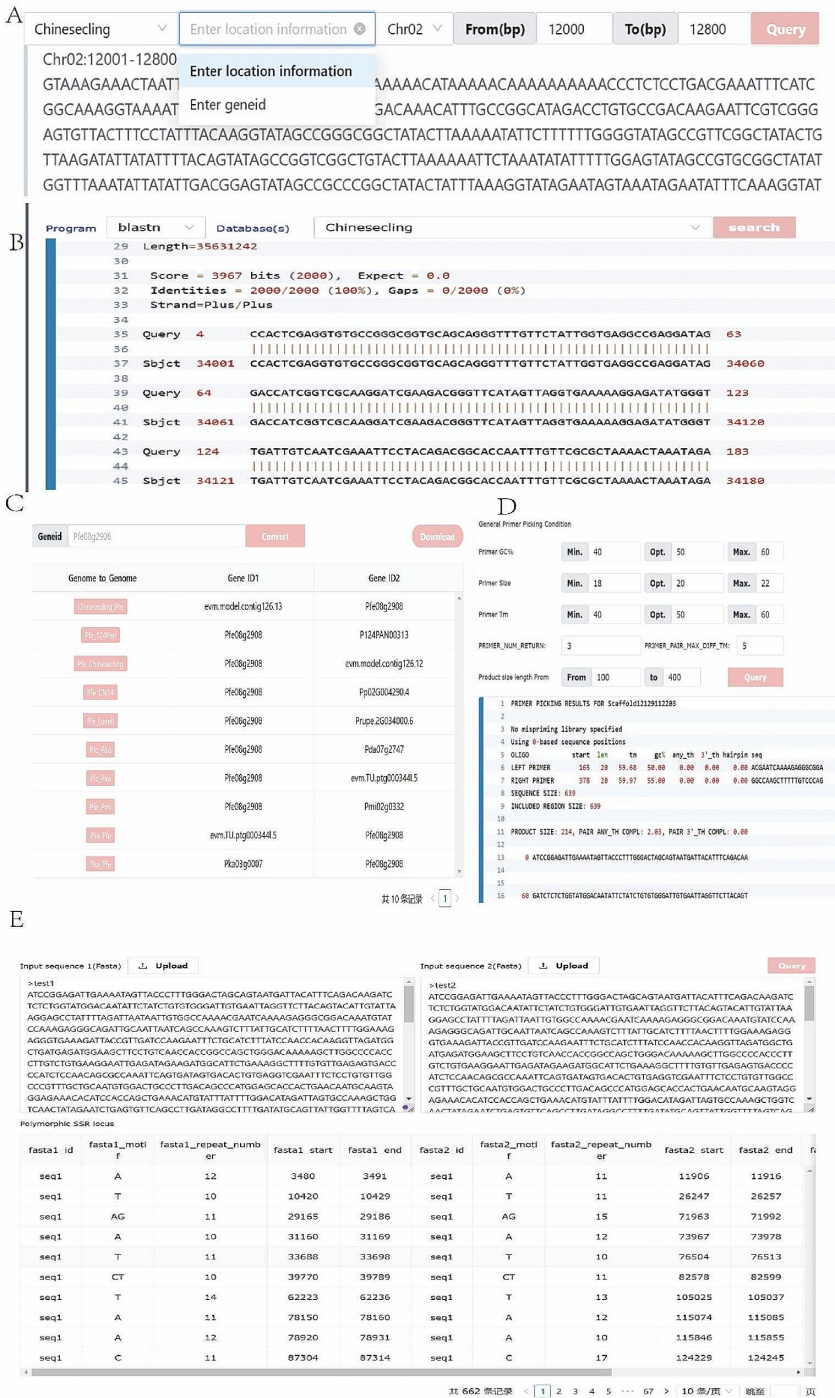
Tools built-in biology tool example. (**A**) Sequence fetch function. (**B**) Sequence alignment function. (**C**) Gene ID conversion. (**D**) Primer design. (**E**) Polymorphism SSR site identification

**Fig. 5:**
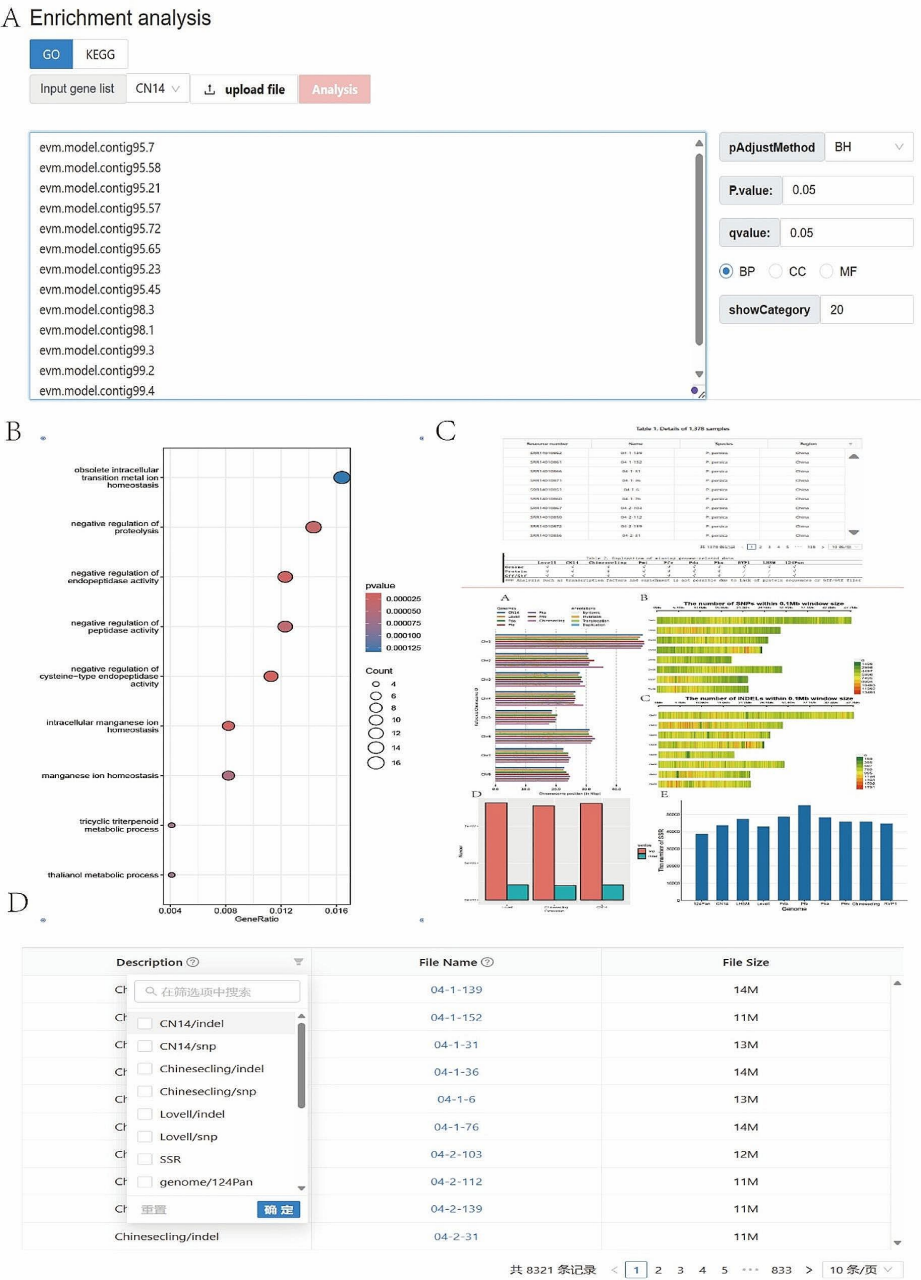
Examples of enrichment analysis and other functional modules. (**A**) and (**B**) represent the enrichment analysis input page and the result display, respectively. (**C**) Overview page. (**D**) Download page

To enhance the functionality of the PPGV site, we have configured several commonly used biology tools in the Tools module, including GetSequence, BLAST, IDConvert, orfFinder, Primerdesign, PolySSR, and EnrichmentAnalysis. In this module, users can not only obtain the corresponding sequences according to the input location information or gene ID (Fig. 4A), but also perform blastn, blastp and TEblast analysis through the alignment function (Fig. 4B). At the same time, through the gene ID converter, homologous genes of different genes can be obtained (Fig. 4C). In addition, primer design and identification of polymorphic SSR sites are also built into the Tools module, and users can enter sequences and parameters and click the submit button to obtain the corresponding results (Fig. 4D, E). It should be noted that for PPGV, all input sequences need to be in Fasta format. Moreover, PPGV can also perform orffinder and KEGG/GO enrichment analysis. As shown in Fig. 5A, after selecting the GO/KEGG enrichment analysis, the user can upload the target gene set or paste it directly into the input box, and then select the more detailed analysis parameters on the right side of the page and click submit, and wait for a short time to download the corresponding analysis results (Fig. 5B).

### JBrowse, download and overview module

Due to the volume of population variation data obtained based on three different reference genomes exceeding 1 Tb, therefore, in the JBrowse module, we only provide the browsing function for genome and GFF files to ensure a good user interaction experience. The Overview module displays a list of all samples and density and quantity statistics graphs related to variations (Fig. 5C). Especially through the download module, users can not only download SNP/SV and INDEL mutation files of all samples, but also obtain genome sequences, protein sequences, gff files, and SSR locus file (Fig. 5D). All these genomic data sets and resources promote the rapid and simple genetic analysis of peach crops.

## Discussion

In recent years, the explosive growth of genome and other sequencing data has promoted the progress of plant database research. More and more databases are being released that integrate massive amounts of data [28–30]. At present, ten peach tree genomes and thousands of resequencing data have been published, however, these data are not yet effectively integrated for deep utilization.

Although some published *Rosaceae* databases contain some peach tree data, these databases are still not comprehensive enough. Such as the famous GDR (https://www.rosaceae.org/) database, which only contains a small amount of peach tree population variation information. The newly released ROFT database contains the transcription and expression information of four Rosaceae plants, including peach trees [31]. In this study, we collected 10 sets of peach genome and 1, 378 samples of resequencing data, and through multiple analysis strategies, tens of millions of variation information including SNP/INDEL/SV were integrated into the PPGV database. At the same time, users can not only easily obtain tandem repeat genes, collinear genes and transposon sequences on the genome, but also query SSR loci and transcription factor sequences through the interactive interface. At the same time, PPGV is equipped with a variety of bioinformatics tools, such as Blast, primer design, and enrichment analysis, which enhance the practicality of the database and will contribute to the genetic breeding research of peach trees.

However, it needs to be acknowledged that some of the genomic protein data and GFF files in this database still need to be supplemented. At present, the genome quality of peach trees still needs to be improved, and a large number of resequencing data are still dominated by next-generation sequencing. In the future, we will continue to update this site with the release of peach tree T2T genome and long-read population sequencing data (this database will integrate the newly released genome and resequencing data every 12 months until a more complete database is released). In conclusion, PPGV will promote the study of peach tree genome and population genome, and contribute to peach tree breeding and fruit improvement.

## Data Availability

All materials and related data in this study are provided in the PPGV database.

## Abbreviations

PPGV: Peach Population Genome Variation
SNP: Single nucleotide polymorphism
INDEL: Insertion-deletion
SV: Structure Variantion
TE: Transposable element

## References

[1] Cao K, Yang X, Li Y, Zhu G, Fang W, Chen C, Wang X, Wu J, Wang L. New high-quality peach (Prunus persica L. Batsch) genome assembly to analyze the molecular evolutionary mechanism of volatile compounds in peach fruits. Plant J. 2021;108(1):281–95. 34309935 10.1111/tpj.15439

[2] Lian X, Zhang H, Jiang C, Gao F, Yan L, Zheng X, Cheng J, Wang W, Wang X, Ye X, et al. De novo chromosome-level genome of a semi-dwarf cultivar of Prunus persica identifies the aquaporin PpTIP2 as responsible for temperature-sensitive semi-dwarf trait and PpB3-1 for flower type and size. Plant Biotechnol J. 2022;20(5):886–902. 34919780 10.1111/pbi.13767 PMC9055816

[3] Cao K, Wang B, Fang W, Zhu G, Chen C, Wang X, Li Y, Wu J, Tang T, Fei Z, et al. Combined nature and human selections reshaped peach fruit metabolome. Genome Biol. 2022;23(1):146. 35788225 10.1186/s13059-022-02719-6 PMC9254577

[4] Zhang A, Zhou H, Jiang X, Han Y, Zhang X. The Draft Genome of a Flat Peach (Prunus persica L. cv. ‘124 Pan’) Provides Insights into Its Good Fruit Flavor Traits. Plants (Basel) 2021, 10(3). 10.3390/plants10030538 PMC7998450 33809190

[5] Verde I, Abbott AG, Scalabrin S, Jung S, Shu S, Marroni F, Zhebentyayeva T, Dettori MT, Grimwood J, Cattonaro F, et al. The high-quality draft genome of peach (Prunus persica) identifies unique patterns of genetic diversity, domestication and genome evolution. Nat Genet. 2013;45(5):487–94. 23525075 10.1038/ng.2586

[6] Yu Y, Guan J, Xu Y, Ren F, Zhang Z, Yan J, Fu J, Guo J, Shen Z, Zhao J, et al. Population-scale peach genome analyses unravel selection patterns and biochemical basis underlying fruit flavor. Nat Commun. 2021;12(1):3604. 34127667 10.1038/s41467-021-23879-2 PMC8203738

[7] Cao K, Zheng Z, Wang L, Liu X, Zhu G, Fang W, Cheng S, Zeng P, Chen C, Wang X, et al. Comparative population genomics reveals the domestication history of the peach, Prunus persica, and human influences on perennial fruit crops. Genome Biol. 2014;15(7):415. 25079967 10.1186/s13059-014-0415-1 PMC4174323

[8] Guan J, Xu Y, Yu Y, Fu J, Ren F, Guo J, Zhao J, Jiang Q, Wei J, Xie H. Genome structure variation analyses of peach reveal population dynamics and a 1.67 mb causal inversion for fruit shape. Genome Biol. 2021;22(1):13. 33402202 10.1186/s13059-020-02239-1 PMC7784018

[9] Li Y, Cao K, Zhu G, Fang W, Chen C, Wang X, Zhao P, Guo J, Ding T, Guan L, et al. Genomic analyses of an extensive collection of wild and cultivated accessions provide new insights into peach breeding history. Genome Biol. 2019;20(1):36. 30791928 10.1186/s13059-019-1648-9 PMC6383288

[10] Yu Y, Fu J, Xu Y, Zhang J, Ren F, Zhao H, Tian S, Guo W, Tu X, Zhao J, et al. Genome re-sequencing reveals the evolutionary history of peach fruit edibility. Nat Commun. 2018;9(1):5404. 30573726 10.1038/s41467-018-07744-3 PMC6302090

[11] Zhao YL, Li Y, Cao K, Yao JL, Bie HL, Khan IA, Fang WC, Chen CW, Wang XW, Wu JL et al. MADS-box protein PpDAM6 regulates chilling requirement-mediated dormancy and bud break in peach. Plant Physiol 2023. 10.1093/plphys/kiad291 PMC10469376 37217835

[12] Xie L, Liu M, Zhao L, Cao K, Wang P, Xu W, Sung WK, Li X, Li G. RiceENCODE: a comprehensive epigenomic database as a rice encyclopedia of DNA elements. Mol Plant. 2021;14(10):1604–6. 34455096 10.1016/j.molp.2021.08.018

[13] Ma S, Wang M, Wu J, Guo W, Chen Y, Li G, Wang Y, Shi W, Xia G, Fu D, et al. WheatOmics: a platform combining multiple omics data to accelerate functional genomics studies in wheat. Mol Plant. 2021;14(12):1965–8. 34715393 10.1016/j.molp.2021.10.006

[14] Wang X-j, Wei Y-f, Liu Z, Yu T, Fu Y-h. Song X-m: TEGR: a comprehensive Ericaceae Genome Resource database1. J Integr Agric 2023.

[15] Gui S, Yang L, Li J, Luo J, Xu X, Yuan J, Chen L, Li W, Yang X, Wu S et al. ZEAMAP, a Comprehensive Database Adapted to the Maize Multi-Omics Era. *iScience* 2020, 23(6):101241. 10.1016/j.isci.2020.101241 PMC7306594 32629608

[16] Zheng T, Li Y, Li Y, Zhang S, Ge T, Wang C, Zhang F, Faruquee M, Zhang L, Wu X, et al. A general model for germplasm-omics data sharing and mining: a case study of SoyFGB v2.0. Sci Bull (Beijing). 2022;67(17):1716–9. 36546052 10.1016/j.scib.2022.08.001

[17] Cui X, Hu M, Yao S, Zhang Y, Tang M, Liu L, Cheng X, Tong C, Liu S. BnaOmics: a comprehensive platform combining pan-genome and multi-omics data from Brassica napus. Plant Commun 2023:100609. 10.1016/j.xplc.2023.100609 PMC10504585 37098652

[18] Luo H, Zhao W, Wang Y, Xia Y, Wu X, Zhang L, Tang B, Zhu J, Fang L, Du Z, et al. SorGSD: a sorghum genome SNP database. Biotechnol Biofuels. 2016;9:6. 26744602 10.1186/s13068-015-0415-8 PMC4704391

[19] An Y, Zhang X, Jiang S, Zhao J, Zhang F. TeaPVs: a comprehensive genomic variation database for tea plant (Camellia sinensis). BMC Plant Biol. 2022;22(1):513. 36324064 10.1186/s12870-022-03901-5 PMC9632082

[20] Chen S, Zhou Y, Chen Y, Gu J. Fastp: an ultra-fast all-in-one FASTQ preprocessor. Bioinformatics. 2018;34(17):i884–90. 30423086 10.1093/bioinformatics/bty560 PMC6129281

[21] Ercolano MR, Di Donato A, Sanseverino W, Barbella M, De Natale A, Frusciante L. Complex migration history is revealed by genetic diversity of tomato samples collected in Italy during the eighteenth and nineteenth centuries. Hortic Res. 2020;7:100. 32637128 10.1038/s41438-020-0322-4 PMC7327043

[22] Cingolani P, Platts A, Wang LL, Coon M, Nguyen T, Wang L, Land SJ, Lu X, Ruden DM. A program for annotating and predicting the effects of single nucleotide polymorphisms, SnpEff. Fly. 2014;6(2):80–92. 10.4161/fly.19695 PMC3679285 22728672

[23] Layer RM, Chiang C, Quinlan AR, Hall IM. LUMPY: a probabilistic framework for structural variant discovery. Genome Biol. 2014;15(6):R84. 24970577 10.1186/gb-2014-15-6-r84 PMC4197822

[24] Zhang RG, Li GY, Wang XL, Dainat J, Wang ZX, Ou S, Ma Y. TEsorter: an accurate and fast method to classify LTR-retrotransposons in plant genomes. Hortic Res 2022, 9. 10.1093/hr/uhac017 PMC9002660 35184178

[25] Ou S, Su W, Liao Y, Chougule K, Agda JRA, Hellinga AJ, Lugo CSB, Elliott TA, Ware D, Peterson T et al. Benchmarking transposable element annotation methods for creation of a streamlined, comprehensive pipeline. Genome Biol 2019, 20(1). 10.1186/s13059-019-1905-y PMC6913007 31843001

[26] Chen C, Chen H, Zhang Y, Thomas HR, Frank MH, He Y, Xia R. TBtools - an integrative toolkit developed for interactive analyses of big biological data. Mol Plant 2020. 10.1016/j.molp.2020.06.009 32585190

[27] Gou X, Shi H, Yu S, Wang Z, Li C, Liu S, Ma J, Chen G, Liu T, Liu Y. SSRMMD: a Rapid and Accurate Algorithm for Mining SSR feature loci and candidate polymorphic SSRs based on assembled sequences. Front Genet 2020, 11. 10.3389/fgene.2020.00706 PMC7398111 32849772

[28] Droc G, Martin G, Guignon V, Summo M, Sempéré G, Durant E, Soriano A, Baurens F-C, Cenci A, Breton C et al. The banana genome hub: a community database for genomics in the Musaceae. Hortic Res 2022, 9. 10.1093/hr/uhac221 PMC9720444 36479579

[29] Su X, Yang L, Wang D, Shu Z, Yang Y, Chen S, Song C. 1 K Medicinal Plant Genome Database: an integrated database combining genomes and metabolites of medicinal plants. Hortic Res 2022, 9. 10.1093/hr/uhac075 PMC9160725 35669712

[30] Yu T, Ma X, Liu Z, Feng X, Wang Z, Ren J, Cao R, Zhang Y, Nie F, Song X. TVIR: a comprehensive vegetable information resource database for comparative and functional genomic studies. Hortic Res 2022, 9. 10.1093/hr/uhac213 PMC9719039 36483087

[31] Li M, Mount SM, Liu Z. Rosaceae Fruit Transcriptome Database (ROFT) – a useful genomic resource for comparing fruits of apple, peach, strawberry, and raspberry. Hortic Res 2023. 10.1093/hr/uhad240 PMC10756754 38162465

